# Rare Papillary Breast Carcinoma Incidentally Discovered After Trauma-Induced Hematoma

**DOI:** 10.7759/cureus.18215

**Published:** 2021-09-23

**Authors:** Becky Li, Jackie Nguyen, Caitlin A Williams, Karina Cardenas, Ihor Pidhorecky

**Affiliations:** 1 Surgery, Nova Southeastern University Dr. Kiran C. Patel College of Allopathic Medicine, Fort Lauderdale, USA; 2 Surgery, Westside Regional Medical Center, Plantation, USA; 3 Surgical Oncology, Westside Regional Medical Center, Plantation, USA

**Keywords:** breast cancer pathology, encapsulated papillary carcinoma, papillary breast tumors, hematoma evacuation, estrogen receptor (er), intracystic papillary carcinoma

## Abstract

Papillary carcinoma of the breast is rare, comprising only 0.5% incidence of all breast cancers. Clinically the disease presents in postmenopausal women as a painless breast lump with possible bloody nipple discharge. Prognosis is favorable due to its slow growth. We present a 61-year-old woman incidentally diagnosed with papillary breast carcinoma after presenting with a trauma-induced hematoma of the right breast. The patient presented to our surgery oncology clinic for persistent right breast swelling secondary to a fall, despite initial incision and drainage (I&D) six weeks prior. She had no history of breast cancer. On presentation, her right breast was distended demonstrating an approximately 20cm ill-defined solid mass with skin changes consistent with a tense hematoma. CT scan demonstrated a large complex cystic and solid breast mass measuring 15.2cmx11.8cmx15.2cm with irregular peripheral solid hyperdense polypoid components. She then underwent a right breast incisional biopsy and hematoma evacuation. Frozen sections of the mass outer cavity wall and papillary projections were consistent with encapsulated papillary carcinoma (EPC). The patient was lost to follow-up and did not obtain definitive treatment. Breast cancer rarely presents as a breast hematoma. However, as in this case, if the hematoma fails to resolve, further investigation is warranted. The prognosis of EPC is excellent when identified and treated appropriately.

## Introduction

Papillary carcinoma of the breast is rare, comprising only 0.5% incidence of all breast cancers [1]. Clinically, the disease presents in postmenopausal women as a painless breast lump with possible hemorrhagic discharge from the nipple [1]. Various risk factors have been identified that contribute to the development of papillary carcinoma. These include increasing age, obesity, family history, alcohol consumption, oral contraceptive use, and hormone replacement therapy [2]. For approximately 50% of patients, these malignant lesions are found in the subareolar region [3]. In this report, we present a 61-year-old woman incidentally diagnosed with papillary breast carcinoma after presenting with a trauma-induced hematoma of the right breast. This case was previously presented as a meeting poster at the 2021 American Medical Student Association (AMSA) Poster Session on March 6, 2021. 

## Case presentation

A 61-year-old woman was referred to the surgical oncology clinic for persistent right breast swelling secondary to a fall on the right chest wall against a solid object approximately seven weeks prior. Her breast swelling had been previously incidentally identified and addressed when the patient was admitted six weeks prior for perforated cholecystitis, requiring hospitalization and cholecystectomy. Following the closure and removal of drapes post-cholecystectomy, an incision and drainage (I&D) of the right breast was performed due to its large fluctuant size. Approximately 100cc of hemorrhagic fluid was drained, which “mostly” decompressed the breast mass per operative report. At that time, fluid was sent only for gram stain and culture, which returned negative for any growth. The swelling did not resolve, prompting her to seek a further evaluation with surgical oncology at our clinic. She denied any family history of breast cancer and never had a mammogram.

Physical exam was significant for massive right breast enlargement due to an approximately 20cm ill-defined solid mass, and skin changes consistent with a tense hematoma. No bleeding, inverted nipple, or nipple discharge were appreciated. The breast was not tender to palpation.

**Figure 1:**
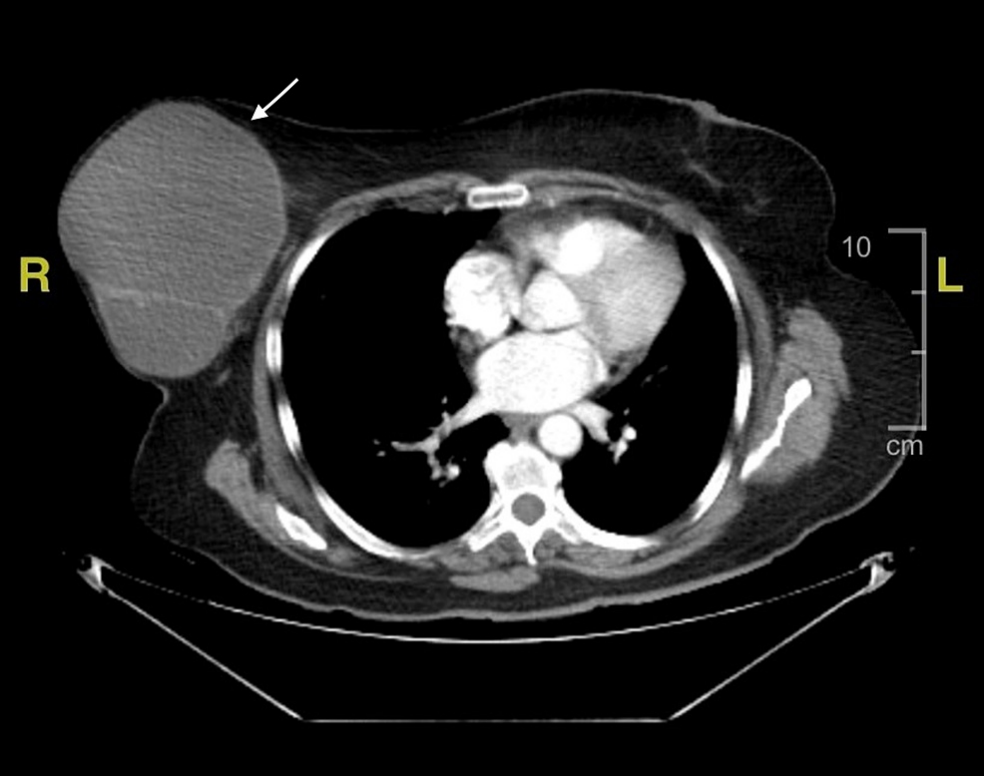
Axial enhanced CT imaging of the thorax. The image demonstrates a right-sided solid mass lesion (white arrow) measuring 15.2cmX11.8cmX 15.2cm. The mass is septated, predominantly cystic, and contains solid, irregularly-shaped polypoid components.

Chest CT demonstrated a large complex cystic and solid breast mass measuring 15.2cmx11.8cmx15.2cm with irregular peripheral solid hyperdense polypoid components (Figure 1). Ultrasound of the right breast showed similar findings consistent with breast imaging-reporting and data system (BIRAD) category IV. According to the radiologist’s impressions, both imaging studies were suspicious for neoplasm versus post-traumatic seroma with hematoma, given her history of prior I&D of the breast, prompting surgical evaluation and evacuation.

The patient consented to a right breast incisional biopsy and hematoma evacuation. The initial circumareolar incision demonstrated that the cavity was tense and contained old blood. The sampled blood was sent for gram stain and culture, which once again returned negative for any growth. On gross examination, the cavity contained multiple papillary projections, which were friable and bled when gently manipulated. Biopsies of the papillary projection and right breast cavity wall were sent to pathology. The papillary projections found inside the cavity were negative for transformation-related protein 63 (P63) and calponin, whereas the cavity wall of the right breast lesion around the ducts was positive for P63 and calponin indicating myoepithelial cell presence. The clinical appearance and immunohistochemistry of these biopsies were most consistent with well-differentiated, low-grade (grade 1), encapsulated papillary carcinoma (EPC). 

**Figure 2:**
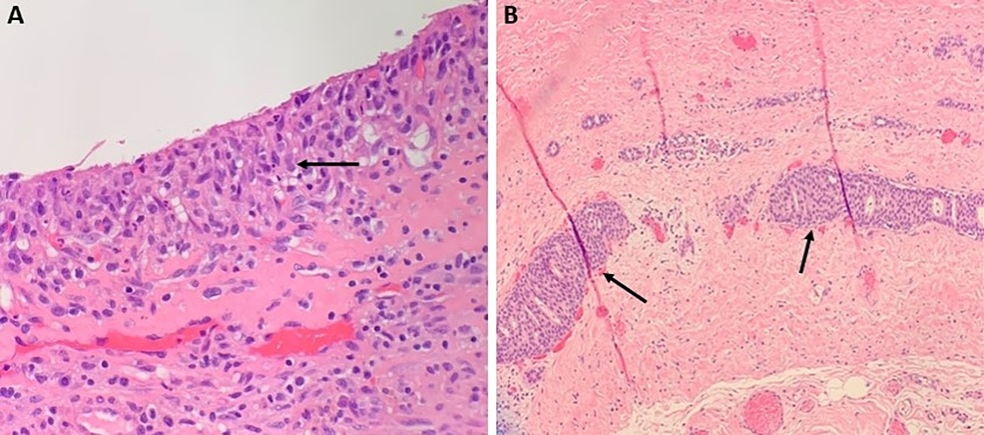
Histopathology of right breast biopsy from the cavity wall. A. Border of the cavity wall demonstrated containing mixed inflammatory infiltrate (histiocytes, neutrophils, lymphocytes) without evidence of carcinoma. B. Two foci of low-grade ductal carcinoma in situ (DCIS) are seen, the greatest linear dimension measuring 2mm.

**Figure 3:**
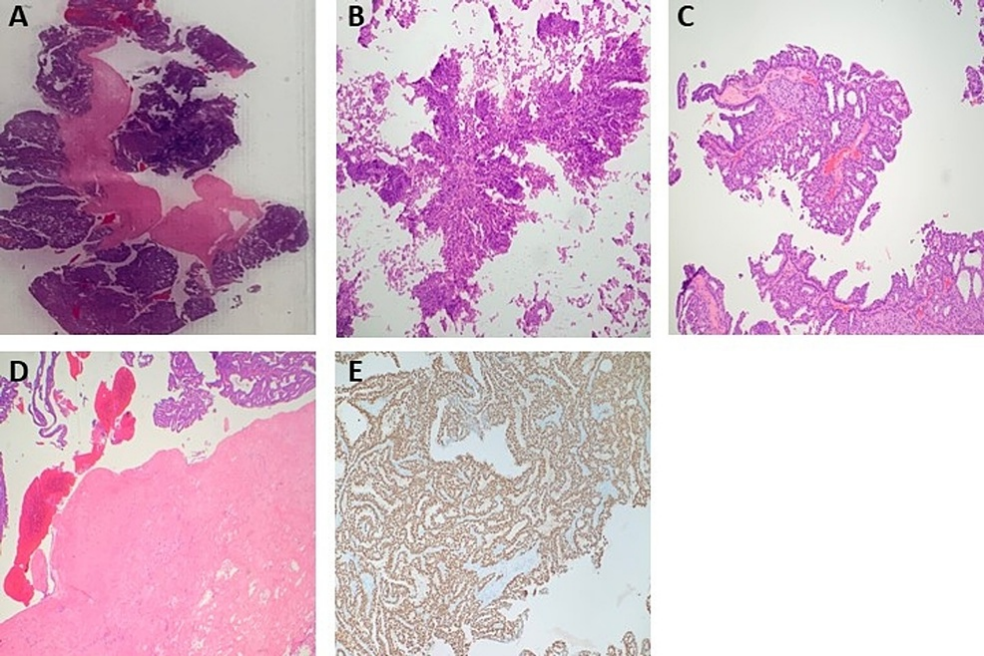
Histopathology of right breast biopsy of papillary projection found in cavity. A & B. Frozen sections containing areas of proliferating homogeneous papillary cells with a thick fibrous capsule, consistent with papillary carcinoma. C. Permanent section demonstrating homogenous papillary proliferation with micropapillary, solid, and cribriform patterns, consistent with papillary carcinoma. D. Permanent section demonstrating homogenous papillary cells completely surrounded by a thick fibrous capsule. There was no evidence of invasion through the capsule, supporting the diagnosis of encapsulated papillary carcinoma. E. Immunohistochemistry staining for the estrogen receptor was diffusely positive.

On the frozen sections of the outer cavity wall biopsy, two focal areas of low-grade cribriform ductal carcinoma in situ (DCIS) were identified as well as a fibrous wall with hemosiderin deposition, chronic xanthogranulomatous inflammation, and cholesterol clefts (Figure 2). The frozen sections of the papillary projections found within the breast cavity demonstrated evidence of sclerosis, ischemic necrosis, and hemorrhage. Notably, there were areas of proliferating homogeneous cells (predominantly papillary) with areas encompassing micropapillary, solid, and cribriform patterns, measuring 6cm collectively (Figure 3). Cells were positive for estrogen-receptor (ER), and negative for basal cytokeratins (CK 5/6). As noted above, immunohistochemistry was negative for P63 and calponin in the papillary projections, demonstrating no evidence of myoepithelial cells in the papillary projections. 

Per pathology, a complete resection was needed to rule out an invasive carcinoma. A mastectomy with sentinel lymph node biopsy was recommended from our surgical standpoint to guide the decision of adjuvant treatment, including the potential for chemotherapy. The patient was lost to follow-up and subsequently did not undergo the recommended mammogram, ultrasound, or definitive surgery.

## Discussion

Blunt trauma to the breast can result in the formation of large hematomas due to the potential for blood pooling into the various tissue planes, such as the subcutaneous fat [4]. Furthermore, trauma to the breast can cause fat necrosis leading to the formation of a tender mass [4]. Fat necrosis and hematomas can mimic the appearance of malignant breast processes on imaging by demonstrating broad septations or wall nodularity [3,5]. However, fat necrosis and hematomas typically resolve over time, thereby distinguishing them from malignant masses [4,6]. Our patient’s initial breast swelling as the result of a fall with subsequent skin changes was consistent with a benign breast process. However, despite the expected development of a hematoma after chest wall trauma, we became concerned for malignancy with the persistence of symptoms after initial drainage despite no previous signs or history indicative of breast cancer. Breast carcinoma presenting as a post-traumatic hemorrhagic cyst is rare, but possible [7]. There are only a few reports of EPC presenting after trauma, and much less so specifying a trauma-induced hematoma or hemorrhagic cyst [8,9]. 

Imaging can assist in diagnosis and may reveal characteristic features of papillary carcinoma. Possible ultrasound findings may include intraductal mass with possible ductal dilatation, intracystic mass, or a solid pattern with an intraductal mass [10]. Papillary carcinoma has been found to have circumscribed round or oval lesions [11]. In contrast to the denser and coarse calcifications of benign papillomas, papillary carcinomas tend to have a non-parallel orientation, echogenic halo, and microcalcifications. Additionally, papillary carcinomas frequently have a larger solid portion with a higher likelihood of intracystic bleeding [1]. Our patient had EPC, also known as intracystic papillary carcinoma. This diagnosis must be differentiated from papilloma, micropapillary DCIS, solid papillary carcinoma, and intraductal papillary carcinoma, which can all have similar clinical and imaging features [12,13].

As a result of the clinically and radiologically ambiguous features, diagnosis of papillary carcinoma can be difficult and often relies on histologic evaluation [14,15]. Histologically, papillary carcinoma characteristically demonstrates papillary proliferation with the growth of fibrovascular stalks surrounded by neoplastic epithelial cells [1]. One feature which distinguishes this malignancy is the absence of myoepithelial cells within papillae [13,14]. Although the lack of myoepithelial cells is a feature that can be in malignancy, it can also be a rare atypical feature of benign papillary lesions [13]. Thus, histological differentials include carcinomas such as solid papillary carcinoma, papillary DCIS, invasive papillary carcinoma, as well as the rare benign apocrine lesion in which a myoepithelial cell layer may be absent [13]. Papillae are also grossly found to be more friable in comparison to those in benign papillomas, likely due to the homogenous epithelium as opposed to the normal heterogeneous composition [15]. Immunohistochemistry shows that malignant growths often fail to express CK5/6 and p63 myoepithelial markers, helping to differentiate them from most benign lesions by confirming lack of myoepithelial cells [16]. Furthermore, EPC is often ER-positive [13,17].

Treatment of papillary carcinoma is dictated by subtype and typically includes excision, either via mastectomy or complete removal of the affected tissue [17]. EPC rarely metastasizes to the lymph nodes at initial presentation and is often successfully treated with partial mastectomy followed by appropriate endocrine therapy [13,17]. In the absence of invasive lesions, the prognosis is excellent, similar to that of DCIS [13]. In our patient, the appearance of DCIS in neighboring tissue does indicate an increased risk of local recurrence [13]. Per National Comprehensive Cancer Network (NCCN) guidelines, systemic adjuvant therapy is considered for those with early-stage breast cancer to lower the risk of breast cancer recurrence [18]. Therapeutic response will differ individually, thus the benefits must be weighed against risks such as toxicity and comorbidity [18]. In hormone-receptor (ER and/or progesterone) positive/human epidermal growth factor receptor 2 (HER2)-negative breast tumors, adjuvant therapy includes endocrine therapy; in those receiving endocrine therapy who are still at high risk of recurrence, adjuvant chemotherapy may be an option [18]. This has been shown to have the greatest benefit in tumors with high-risk features, such as ≥4 positive lymph nodes [18]. Indecision, whether to use adjuvant chemotherapy, can be mitigated through gene expression analysis, which can help predict the effectiveness of adding chemotherapy to the treatment regimen [18].

## Conclusions

Breast cancer rarely presents as a breast hematoma. There are only a few reported cases of EPC presenting secondary to trauma. EPC presenting secondary to trauma-induced hematoma is rarer. Although a history of trauma to the breast can suggest a more benign etiology of hematoma, persistent symptoms should raise clinical suspicion and prompt further diagnostic workup. Imaging demonstrating solid components should prompt an open biopsy. In our case, the biopsy demonstrated EPC suggesting the traumatic incident likely exacerbated an already fragile tumor and caused a tense hematoma. Histologically, EPC is characterized by a cystically dilated duct surrounded by a fibrous capsule with intraluminal arborization of the fibrovascular stroma covered by atypical epithelium with low or intermediate nuclear grade with no evidence of necrosis and rare mitoses. The prognosis of EPC is excellent once detected, making early diagnosis crucial.
